# Mixed-reality-based education with holographic support before and during ERCP-related procedures

**DOI:** 10.1055/a-2678-0183

**Published:** 2025-08-20

**Authors:** Yuki Tanisaka, Shomei Ryozawa, Takao Itoi, Masafumi Mizuide, Akashi Fujita, Ryuichi Watanabe, Maki Sugimoto

**Affiliations:** 1 183786 Department of Gastroenterology, Saitama Medical University International Medical Center, Hidaka, Japan; 2 13112 Department of Gastroenterology and Hepatology, Tokyo Medical University, Tokyo, Japan; 3 Innovation Lab, Teikyo University Okinaga Research Institute, Tokyo, Japan

Endoscopic biliary drainage by endoscopic retrograde cholangiopancreatography (ERCP) in patients with malignant biliary obstruction requires a thorough understanding of the biliary anatomy, as well as accurate identification of the appropriate drainage area. However, achieving this level of understanding in trainee education can be challenging when relying on two-dimensional fluoroscopic images. Mixed reality (MR) is a technology that merges real-world and virtual elements in real time to create an integrated environment [Bibr LI_LiteratureBookmark_1] . By wearing a dedicated head-mounted display and hand controllers (Meta Quest 3; Meta, USA), users can view holograms, which are computer-generated three-dimensional (3D) graphic models, from their desired perspective ( [Fig FI_Ref205533950] ). It has been reported that MR with holographic support is feasible for pancreatobiliary endoscopic procedures [Bibr LI_LiteratureBookmark_2] [Bibr LI_LiteratureBookmark_3] [Bibr LI_LiteratureBookmark_4] . We report a case where ERCP was carried out in a patient with hilar bile duct cancer, in which MR-based education with holographic support was feasible.

**a FI_Ref205533950:**
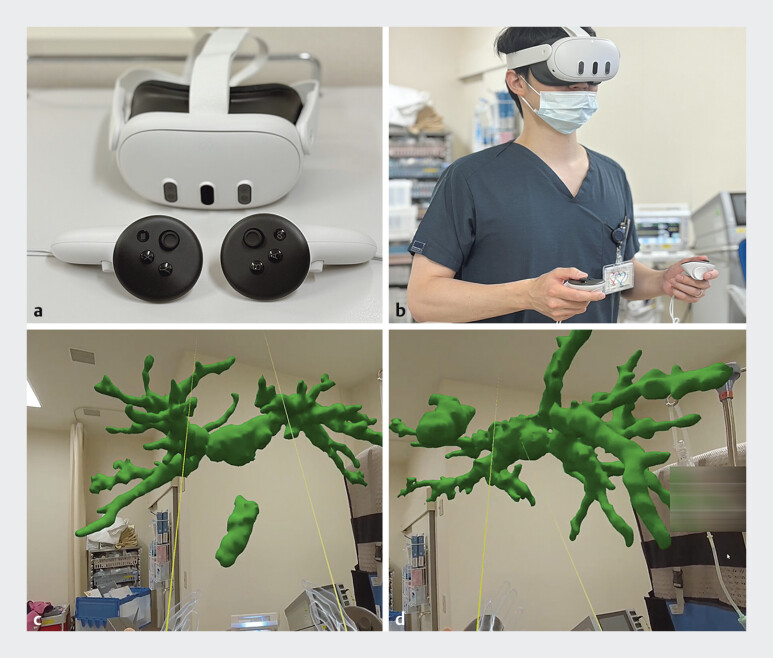
Mixed-reality-based education with holographic support. A dedicated head-mounted display and hand controllers. **b** A doctor wearing a head-mounted display and using hand controllers. **c** A computer-generated three-dimensional (3D) graphic model seen through the head-mounted display. **d** The 3D model is rotatable.

A 68-year-old man with resectable hilar bile duct cancer was referred to us ( [Fig FI_Ref205533954] ). After discussion with surgeons, we planned to perform ERCP and biliary drainage of the left intrahepatic bile duct ( [Media ME_Ref205533969] ). 3D polygon data of the bile duct was created from computed tomography using Synapse Vincent (Fujifilm Medical Co., Ltd., Japan) and Holoeyes MD software (Holoeyes Inc., Japan) ( [Fig FI_Ref205533958] ). Before the procedure, trainees received tuition from experts on the patient’s biliary anatomy and the drainage area while using MR with holographic support ( [Fig FI_Ref205533961] ). Furthermore, they also received tuition while viewing MR alongside fluoroscopic findings during the procedure ( [Fig FI_Ref205533964] ). Since the MR and fluoroscopic findings were comparable, this helped trainees’ understanding of the procedure. Finally, biliary drainage was performed in the area explained by the experts to the trainees before the procedure using MR.

**Figure FI_Ref205533954:**
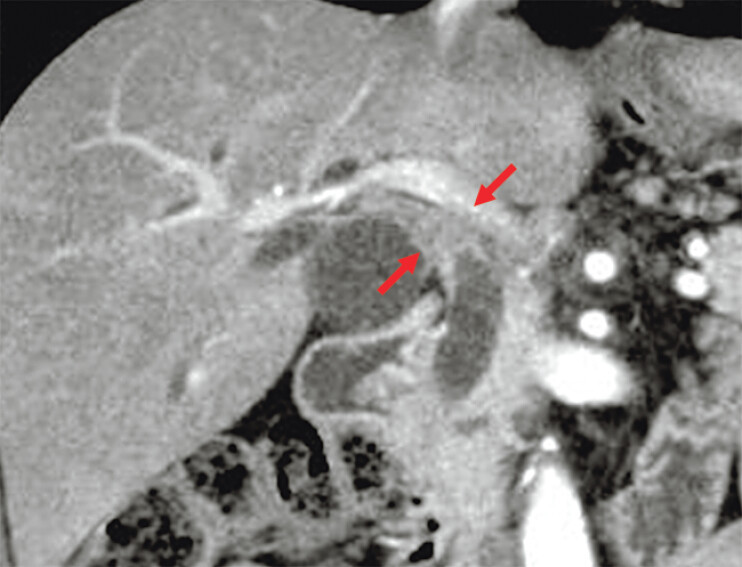
Computed tomogram showing the biliary stricture caused by bile duct cancer in the hilar bile duct of a 68-year-old man (arrows).

**Figure FI_Ref205533958:**
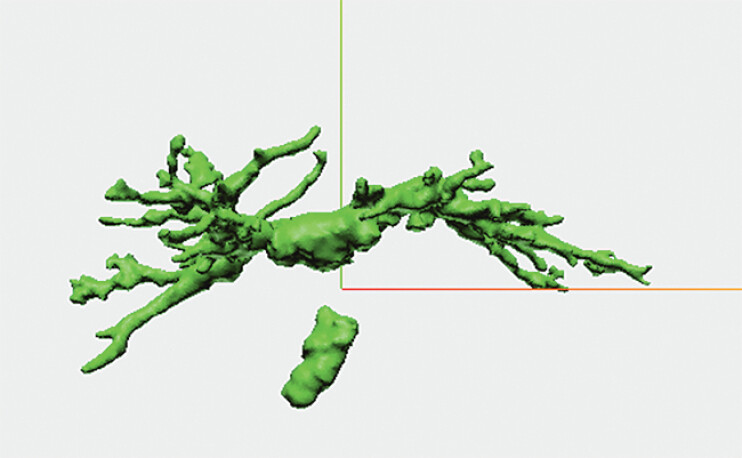
Computed tomographic imaging converted into 3D polygon data.

**Figure FI_Ref205533961:**
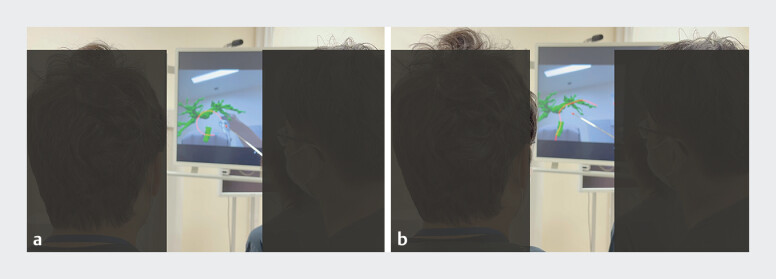
Before the procedure, trainees received tuition from experts on the patient’s biliary anatomy and the drainage area, using mixed reality with holographic support.

**Figure FI_Ref205533964:**
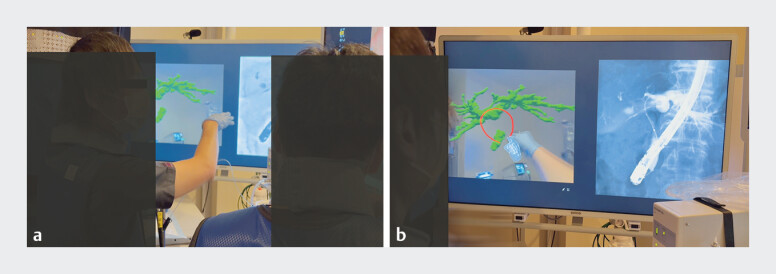
Trainees also received tuition while viewing mixed reality with holographic support alongside fluoroscopic imaging during the procedure.

Mixed-reality-based education with holographic support before and during endoscopic retrograde cholangiopancreatography–related procedures.

MR-based education with holographic support both before and during ERCP was feasible. This approach may represent an innovative educational technology for pancreatobiliary endoscopists.

Endoscopy_UCTN_Code_TTT_1AR_2AB

## Notes

Endoscopy E-Videos https://eref.thieme.de/e-videos
*E-Videos* is an open access online section of the journal *Endoscopy* , reporting on interesting cases and new techniques in gastroenterological endoscopy. All papers include a high-quality video and are published with a Creative Commons CC-BY license. Endoscopy E-Videos qualify for HINARI discounts and waivers and eligibility is automatically checked during the submission process. We grant 100% waivers to articles whose corresponding authors are based in Group A countries and 50% waivers to those who are based in Group B countries as classified by Research4Life (see: https://www.research4life.org/access/eligibility/ ). This section has its own submission website at https://mc.manuscriptcentral.com/e-videos .
